# Optimization of Gene Gun-Mediated Transient Transformation and Explant Suitability in Coconut

**DOI:** 10.3390/plants15010150

**Published:** 2026-01-04

**Authors:** Mingjun Ma, Hanlu Su, Hao Nie, Xiaomeng Fang, Saeed Rauf, Saira Batool, Yin Min Htwe, Dapeng Zhang, Peng Shi, Zhiying Li, Qun Yu, Xiangman He, Yong Wang

**Affiliations:** 1 State Key Laboratory of Tropical Crop Breeding/Sanya Research Institute/Coconut Research Institute, Chinese Academy of Tropical Agricultural Sciences, Wenchang 571339, China; mmjyxh@163.com (M.M.); 13905483918@163.com (H.S.); niehao344@163.com (H.N.); fangxiaomeng222@163.com (X.F.); saeedbreeder@hotmail.com (S.R.); sairabatool0799@outlook.com (S.B.); yinminhtwemgk@gmail.com (Y.M.H.); zhangdp@catas.cn (D.Z.); ship@catas.cn (P.S.); lizhiyingalien@gmail.com (Z.L.); yuqun1998@gmail.com (Q.Y.); 18389739413@163.com (X.H.); 2 College of Horticulture and Forestry Sciences, Huazhong Agricultural University, Wuhan 430070, China; 3 Department of Plant Breeding & Genetics, College of Agriculture, University of Sargodha, Sargodha 40100, Pakistan

**Keywords:** coconut, gene gun transformation, biolistic delivery, transient expression, explant

## Abstract

Coconut genetic improvement remains challenging due to low regeneration efficiency and limited transformation success. We optimized major components of a gene gun-mediated transient transformation system and evaluated explant types to support future establishment of a stable transformation pipeline. Three reporter genes (eGFP, *GUS*, and RUBY) were compared in coconut callus, and eGFP was selected as the most suitable due to its strong and non-destructive fluorescence. Background interference in GUS staining was reduced by adjusting the methanol–GUS ratio to 4:10. Single-factor optimization using callus tissue identified 0.4 M mannitol, 300–500 μg gold particles, 1.5 μg plasmid DNA, a 6.5 cm target distance, and 7 MPa pressure as effective parameters for biolistic delivery. Among the callus types, spongy callus showed strong transient eGFP expression but displayed loose and watery morphology consistent with non-embryogenic callus. In contrast, crumbly and smooth callus exhibited compact structures previously associated with embryogenic competence, although transient expression levels were lower. Among differentiated tissues, germinated zygotic embryo plumules and distal young leaflets exhibited moderate transient expression, supporting their suitability as transformation targets. These findings provide practical guidance on reporter selection, parameter refinement, and explant choice for future establishment of an efficient genetic transformation system in coconut.

## 1. Introduction

Coconut (*Cocos nucifera* L.) is a key economic crop in the Indo-Malaya region, Africa, and the Pacific Islands, with a wide range of uses in both cooking and non-cooking applications. It is often called “the tree of life” because of its numerous culinary and non-culinary purposes in many parts of the world [1]. Its culinary uses include meat (fresh, dried), cream, milk, water, oil, and nutraceuticals. The tree holds aesthetic value and is used for land beautification, while its economic benefits serve a dual purpose in tropical island economies. The elegance of the coconut is often seen as resembling the beauty of a woman in many cultures. It is a highly diverse crop with two main types of cultivars (tall and dwarf) and their hybrids, tall × dwarf [2,3,4]. Each type has distinct characteristics, adaptability, and limitations. However, its cultivation faces significant challenges from various biotic and abiotic stresses, hindering its expansion in many regions [5,6]. Traditional breeding methods are also limited by long generation times.

Genetic engineering, particularly through the overexpression of genes that confer resistance to biotic and abiotic stresses, holds great promise for mitigating yield losses and improving adaptability to suboptimal environmental conditions. CRISPR/Cas9 gene-editing technology also offers a precise method for modifying traits, such as susceptible loci, preventing premature fruit drop, improving fruit quality, and reducing photo-thermal sensitivity [7,8]. Knockout and overexpression lines have also been used in functional genomics to study the physiological functions of a specific gene, its regulatory pathway, and its interactions with the environment [9]. This can help understand how the species responds under certain environmental conditions, and the insights gained can be applied in future breeding programs.

Coconut is a recalcitrant species with inherently low regeneration and transformation capacity. In addition, genetic improvement through conventional breeding is slow and inefficient due to the long juvenile phase and high heterozygosity. In vitro strategies such as transgene overexpression and CRISPR/Cas-based genome editing provide more rapid routes for trait enhancement, including disease resistance, reduced plant height, shortened juvenile stage, lodging resistance, and improved nut and kernel quality, while also enabling the clonal fixation of hybrid vigor. However, somatic embryogenesis in coconut remains highly challenging. Callus induction typically requires several months of culture and is strongly influenced by explant type, resulting in variable regenerative responses and low overall transformation efficiency [10,11]. A range of explants—including plumules, ovaries, and inflorescence tissues—have been evaluated with differing abilities to produce somatic embryos [11,12,13,14], yet comparative analyses of their transformation responsiveness are still limited. Moreover, transformation efficiency in palms is generally low and often inconsistent across studies [8,15]. Although our group recently achieved CRISPR/Cas-mediated genome editing in coconut protoplasts with a transformation efficiency of ~4.02% [8], further improvements are needed. Optimization of reporter gene systems, refinement of gene gun parameters, and systematic evaluation of explant suitability remain critical steps toward establishing an efficient transformation platform for coconut.

Transformation approaches can broadly be categorized as biological or physical approaches. Among physical approaches, particle bombardment (gene gun) is widely used, whereby high-speed microparticles deliver exogenous DNA into target cells [16]. This technique has facilitated gene introduction in tropical species, including those carrying dwarfing or disease-resistance traits. In coconut, transient expression of green fluorescent protein (GFP) has previously been achieved through a combination of *Agrobacterium*-mediated transfer, biolistic bombardment, and vacuum infiltration [15]. Nevertheless, overall transformation remains inefficient due to the reliance on somatic embryogenesis, which is slow and frequently problematic in coconut [10,17]. Agrobacterium-mediated transformation is particularly prone to contamination and often induces tissue browning, while bombardment can cause cellular damage. As a result, the vast majority of transformed cells are lost during regeneration, with current embryogenesis-based methods achieving successful plant recovery from only about 1% of transformed material.

Reporter genes such as eGFP, *GUS*, and RUBY have been used to visualize transient transformation across a wide range of explants and calli. These reporter genes, especially eGFP and RUBY, offer benefits such as early detection, non-invasive monitoring, and real-time visualization of transformed explants [18,19].

However, detecting reporter genes can be complicated by factors such as background fluorescence or staining, which may lead to false positives, as well as photobleaching and limited tissue penetration. These issues make it difficult to accurately assess transformation success, particularly in recalcitrant species like coconut [20]. Additionally, assays for visualizing reporter genes, such as *GUS*, can damage explants or calli. Therefore, it is essential to compare various reporter genes and select the most suitable one to assess transformation efficiency in coconut.

Different explants (leaf blades, embryonic inflorescence, zygotic embryo) and calli (embryogenic and non-embryogenic) show variability in their regeneration and transformation potential. The regenerative capacity and transformation efficiency of each explant type are influenced by factors such as developmental stage, cellular activity, and responsiveness to plant growth regulators. Additionally, it is important to perform histological analyses of the explant’s physiological responses after transformation. These analyses help identify the underlying factors that contribute to successful transformation. Understanding physiological and cellular responses helps optimize transformation protocols and increase overall success rates.

There is an ongoing need to optimize protocols for the transformation and subsequent handling of plant material to address these challenges [15]. Experiments were conducted at the Coconut Research Institute in Sanya to explore various techniques to enhance transformation efficiency using the biolistic gene gun. The studies were conducted to determine transient eGFP expression and to compare it with that of other reporter genes, such as GUS and RUBY. Biolistic gene gun parameters were also compared and selected to fine-tune the transformation procedure. Additionally, comparative experiments were performed using different explants (calli, immature rachilla, leaves, and zygotic embryos) to assess transformation efficiency with various visual and selectable markers. The findings from this research are expected to significantly enhance the success rate of coconut genetic transformation, providing valuable insights for biotechnologists working to insert or modify the gene of interest.

## 2. Results

### 2.1. Selection of Reporter Genes

**Figure 1:**
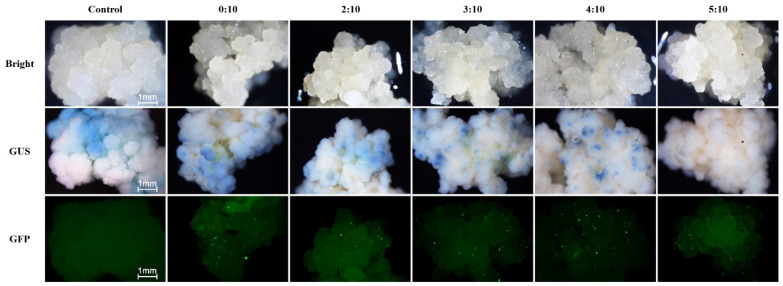
Optimization of GUS staining conditions to reduce endogenous GUS-like background activity in coconut callus tissue. Coconut callus was subjected to GUS histochemical staining with different methanol–GUS dye ratios (0:10, 2:10, 3:10, 4:10, and 5:10) for 12 h at 37 °C. **Top row**: Bright-field images showing general callus morphology. **Middle row**: GUS staining results. The non-transformed control (0:10) shows weak blue coloration due to endogenous GUS-like activity. Increasing methanol concentration (4:10) effectively suppressed endogenous staining while maintaining transgene-derived GUS signal in bombarded calli. **Bottom row**: Corresponding eGFP fluorescence confirms transient transformation events. Scale bars = 1 mm.

In this study, the transformation efficiency of three reporter genes (GUS, eGFP, and RUBY) was evaluated in coconut callus after bombardment with their respective plasmids using a gene gun. The P7 vector carried both the eGFP and *GUS* genes. GUS activity was optimized using various methanol-to-GUS dye ratios. The control (CK) (non-transformed calli) exhibited uneven blue surface areas, indicating endogenous *GUS* activity in coconut callus tissue (Figure 1). On the other hand, experiments demonstrated that GUS staining was dependent on the ratio of methanol to GUS dye. Low methanol concentrations were insufficient to adequately remove background staining, while excessive methanol concentrations prevented proper staining of the transformation gene. The optimal ratio of 4:10 methanol to GUS dye effectively eliminated the background blue caused by endogenous GUS activity in coconut callus tissue, thereby maintaining expression of the transformation gene (Figure 1).

**Figure 2:**
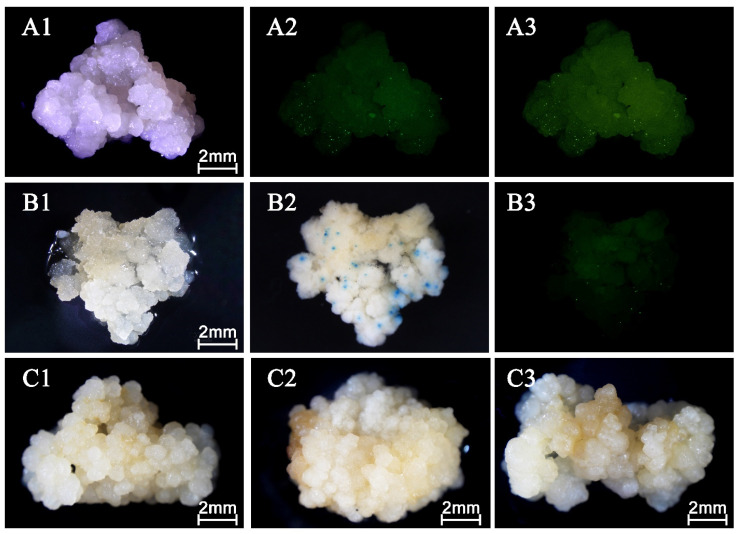
Transformation of coconut palm callus with plasmids encoding various reporter genes (eGFP, *GUS*, and RUBY). (**A1**–**A3**) P7 plasmid (carrying eGFP and *GUS*) bombarded into the coconut callus. Images were taken 24 h after bombardment. (**A1**) Bright-field view. (**A2**) eGFP fluorescence under 480 nm blue excitation. (**A3**) Merged bright-field and GFP fluorescence image showing clear eGFP-expressing cells. (**B1**–**B3**) Same P7 plasmid used for GUS/eGFP co-expression. Images taken after 12 h GUS staining. (**B1**) Callus before GUS staining. (**B2**) GUS staining showing blue precipitates. (**B3**) GFP fluorescence image of the same GUS-stained tissue, illustrating the correspondence between GFP-positive fluorescent spots and GUS-positive blue spots. (**C1**–**C3**) Comparison of RUBY reporter treatments using P2 plasmid. All images taken 72 h after bombardment. (**C1**) Negative control—no gold particles and no plasmid (unbombarded). (**C2**) Gold particles only (empty particles, no plasmid) to distinguish wounding effects from reporter expression. (**C3**) P2 plasmid (RUBY) carried on gold particles. Only slight browning was seen, with no visible RUBY red pigmentation. Scale bars = 2 mm.

Green fluorescence was clearly detected 24 h after bombardment under blue light excitation (Figure 2(A1–A3)). The fluorescent spots showed well-defined boundaries in both the fluorescence and merged images (Figure 2(A2,A3)), confirming successful transient expression of the eGFP reporter gene in coconut callus. In calli transformed with the dual-reporter plasmid, the GFP fluorescence pattern (Figure 2(B3)) corresponded closely with the GUS-positive blue spots observed after staining (Figure 2(B2)). However, adjacent GUS-positive regions tended to merge, forming clusters that made individual spot counting difficult, and the staining procedure itself caused tissue necrosis, which adversely affected subsequent regeneration.

For the RUBY reporter, stereomicroscopic observation at 72 h showed only slight browning of the callus surface without the development of visible red pigmentation (Figure 2(C2,C3)), in contrast to the untreated control (Figure 2(C1)). The absence of detectable red coloration suggests low compatibility of the RUBY system with coconut callus tissue.

Overall, these comparisons indicate that eGFP provides the clearest, most reliable, and least destructive reporter signal, making it the most suitable reporter gene for gene gun–mediated transformation in coconut.

### 2.2. Optimization of Gene Gun Parameters

**Figure 3:**
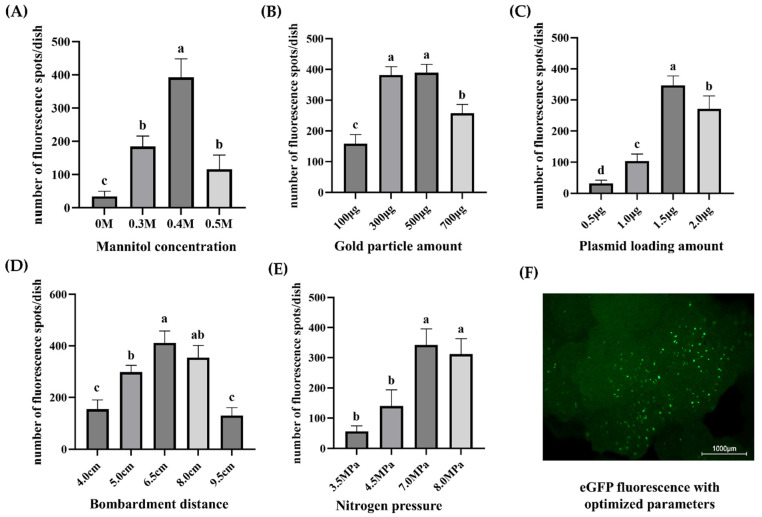
Effects of various biolistic parameters on transient eGFP expression in coconut callus (**A**–**E**). Single-factor evaluation of mannitol concentration (**A**), gold particle amount per bombardment (**B**), plasmid loading amount (**C**), Bombardment distance (**D**), and nitrogen pressure (**E**). For each treatment, approximately 35–40 callus pieces were arranged on a 9 cm Petri dish, and three plates were analyzed independently per parameter. Transient transformation efficiency was quantified as the average number of eGFP fluorescent spots per plate, recorded 24 h after bombardment. (**F**) Representative eGFP fluorescence image obtained under the optimized biolistic conditions: 0.4 M mannitol pre-treatment, 300 µg gold particles per bombardment, 1.5 µg plasmid DNA, 6.5 cm shooting distance, and 7 MPa nitrogen pressure. Scale bars = 1000 µm. Data are presented as mean ± SD (n = 3). Different lowercase letters above bars indicate statistically significant differences among treatments within each parameter based on the LSD test (*p* < 0.05).

The results of optimizing various parameters related to the gene gun transient transformation revealed that 0.4 M mannitol was the optimal concentration of the hyperosmotic solution for pre-treatment (Figure 3). Gene gun parameters were optimized by testing various treatments within each parameter. The treatment that displayed the highest number of fluorescent spots per dish demonstrated the transient transformation efficiency. The highest transient transformation efficiency was achieved using the following parameters of the gene gun: 500 µg of gold particles, 1.5 μg of plasmid per bombardment, a 6.5 cm distance between the carrier membrane and the sample, and a nitrogen pressure of 7 MP. These parameters resulted in the highest number of eGFP fluorescence spots per dish (Figure 3). Although the 500 µg of gold particles had the highest transient transformation efficiency, it was statistically similar to 300 µg, which could also be used to achieve efficient gene gun-mediated transient transformation. Thus, these optimized conditions were established as the preliminary optimal transformation system for coconut genetic transformation, providing a basis for subsequent material screening.

When two treatments produced statistically similar spot numbers (e.g., 300 µg vs. 500 µg gold particles), the final parameter was selected based on both efficiency and practical considerations, including reduced tissue damage, lower material use, and greater consistency across replicates.

### 2.3. Selection of Explant Type

#### 2.3.1. Callus

**Figure 4:**
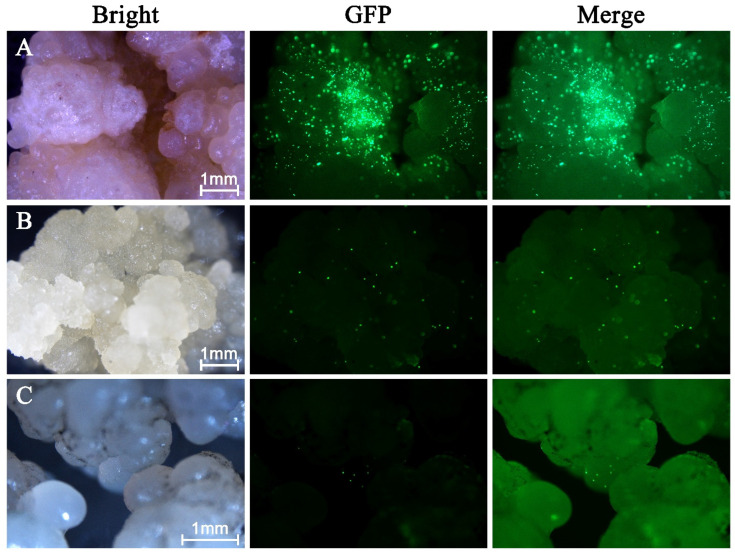
Transient eGFP fluorescence observed in coconut callus tissues of different morphological types. (**A**) Spongy callus showing the highest number of fluorescence spots. (**B**) Crumbly callus with moderate fluorescence. (**C**) Smooth callus with low fluorescence. Scale bars = 1 mm.

Fluorescence analysis of plasmid P13 in the calli (spongy, crumbly and smooth) revealed that the sponge callus had the highest number of fluorescent spots (Figure 4A). The crumbly callus displayed fewer fluorescent spots than the spongy callus (Figure 4B). The smooth callus showed only a small number of fluorescent spots, indicating lower transformation efficiency (Figure 4C). Overall, these results demonstrate that the transformation efficiency of coconut embryogenic calli using gene gun technology remains low.

#### 2.3.2. Zygotic Embryos

**Figure 5:**
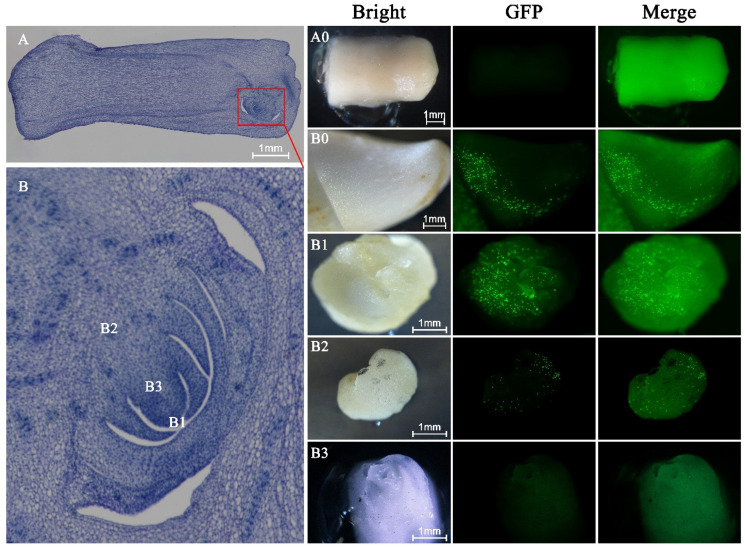
Transient eGFP expression in zygotic embryos and excised plumule tissues following gene gun bombardment. (**A**) Non-germinated zygotic embryos. (**A0**) No detectable GFP fluorescence was observed after bombardment. (**B**) Germinated zygotic embryos and excised plumule tissues. (**B0**) Germinated embryos with intact plumules exhibited GFP fluorescence, which was restricted to the basal region of the plumule. (**B1**) The excised plumule tip showed strong transient GFP expression, although subsequent growth remained limited. (**B2**) The basal portion of the plumule displayed callus formation and weak GFP fluorescence signals. (**B3**) The exposed meristematic region of the plumule, obtained by carefully removing the outer embryonic layer, showed no detectable GFP fluorescence. All images were captured 48 h after bombardment under blue light excitation using a stereo fluorescence microscope (Nikon, Tokyo, Japan). Scale bars = 1 mm.

The P13 vector was bombarded into zygotic embryos and their excised portions. GFP transient expression indicated that non-germinated embryos did not show any fluorescent signals on their surfaces (Figure 5(A0)), whereas germinated embryos exhibited numerous GFP spots on the tender plumule (Figure 5(B0)). However, the fluorescent areas were restricted to the basal region of the plumule, and these transformed tissues eventually necrosed, preventing continued growth and regeneration.

To improve both transformation and subsequent regeneration, the plumule was excised into different tissue types with distinct developmental potentials. Bombardment of the plumule tip resulted in higher transient GFP expression (Figure 5(B1)). However, some transformed regions gradually developed into sponge-like tissues characterized by a loose, porous structure, which corresponds to non-embryogenic callus. These tissues failed to undergo sustained proliferation or somatic embryogenesis, indicating limited regenerative competence despite successful DNA delivery.

The basal portion of the plumule was capable of callus formation and also showed GFP signals after bombardment (Figure 5(B2)), but with clearly lower transformation intensity compared to the apical tissue. In contrast, bombardment of the exposed meristematic region of the plumule, obtained by carefully removing the outer embryonic layer, did not yield detectable GFP fluorescence (Figure 5(B3)).

#### 2.3.3. Rachilla

**Figure 6:**
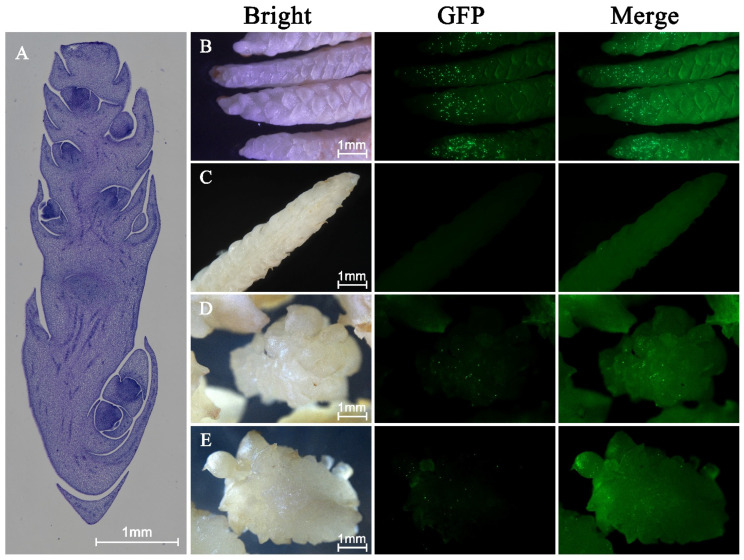
Transient eGFP expression in immature coconut rachilla following gene gun bombardment. (**A**) Longitudinal section of an immature rachilla. The dark blue region corresponds to the floret sites, which are enriched in stem cells and serve as key locations for callus induction. (**B**) GFP fluorescence detected on the surface of freshly excised immature rachilla 48 h after bombardment, indicating the feasibility of transient DNA delivery in exposed outer tissues. (**C**) Longitudinally sectioned rachilla immediately after bombardment showed no detectable GFP fluorescence, likely due to cell death at the cut surface forming a barrier to plasmid penetration. (**D**) Pre-cultivated intact rachilla (10 days prior to bombardment) exhibited weaker GFP signals compared with freshly excised tissue, suggesting limited accessibility of meristematic regions beneath the scales. (**E**) Pre-cultivated longitudinal sections showed only minimal and scattered GFP fluorescence, despite partial regeneration of cut surfaces during culture. All fluorescence images were captured under blue light excitation using a stereo fluorescence microscope. Scale bars: 1 mm.

The rachilla consists of a central axis, florets, and protective scales. In the longitudinal section, the dark blue regions mark the floret sites enriched in stem cells, which are the main sites where callus formation typically occurs (Figure 6A).

For transient transformation, all rachilla samples were collected at the same developmental stage and subjected to identical preculture conditions unless otherwise stated. Immature rachilla segments (approximately 2–3 cm in length) were excised and surface-sterilized, then precultured on Y3 basal medium supplemented with 30 g/L sucrose, 120 mg/L 2,4-D, and 2.5 g/L activated charcoal, solidified with 4 g/L phytagel. Preculture was conducted for 10 days under dark conditions at 28 ± 1 °C, which is consistent with standard conditions for coconut inflorescence-derived callus induction.

Two types of rachilla tissues were used for gene gun bombardment: (i) the intact outer surface of freshly excised immature rachilla, and (ii) the exposed internal tissues obtained by longitudinal sectioning along the rachilla axis.

Following preculture, tissues were transferred to hyperosmotic medium containing 0.4 M mannitol for 6 h prior to bombardment, as described in Section 4.4, and were bombarded using the optimized biolistic parameters. After bombardment, rachilla samples were incubated on the same hyperosmotic medium for an additional 16 h in darkness at 28 °C, followed by transfer to recovery medium for 24 h.

Fluorescence imaging showed multiple GFP-expressing spots on the surface of the immature rachilla (Figure 6B), confirming that gene gun delivery into this tissue is feasible. The protective scales covering the rachilla obstruct plasmid delivery into the floral primordia, thereby limiting effective transformation of inner tissues.

Longitudinal sections of the rachilla were prepared to expose the internal tissues to gene gun bombardment. However, no GFP fluorescence was detected in the cross-sectional areas (Figure 6C). To further evaluate whether tissue recovery could improve transient transformation responsiveness, both intact and longitudinally sectioned rachilla were precultured for 10 days prior to bombardment. After preculture, only weak fluorescence was observed on the rachilla surface and minimal or no signal was detected in longitudinal sections (Figure 6D,E). Based on the eGFP transient expression outcomes, the rachilla can serve as a potential recipient for gene gun transformation. However, efficient DNA delivery into the floral primordia beneath the protective scales remains limited and requires further optimization.

#### 2.3.4. Leaves

**Figure 7:**
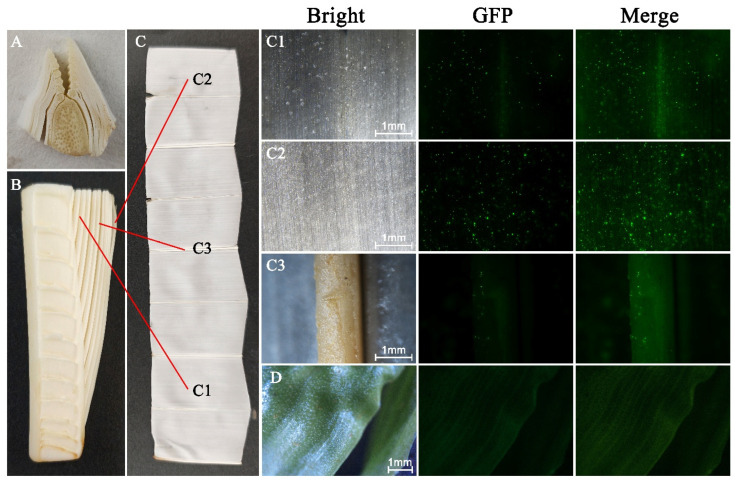
Transient eGFP expression in different regions of coconut young leaves after gene gun bombardment. (**A**) Cross-section of a young leaf. (**B**) Longitudinal section of a young leaf. (**C**) Leaflet regions examined: (**C1**) distal leaflet region away from the leaf axis, (**C2**) proximal leaflet region near the leaf axis, and (**C3**) leaflet vein region. (**D**) Green mature leaf. Distal leaflets showed stronger fluorescence signals and better tissue tolerance to bombardment compared with other leaf regions. Scale bars = 1 mm.

Leaves serve as potential explant sources for inducing callus tissue, offering relatively easy material acquisition for genetic transformation. In this study, several types of leaves were selected based on their position on young coconut trees (Figure 7A–C). The leaves, including those near the leaf axis, distal from the axis, vein portions, and green leaves of photoperiodically mature seedlings, were selected for transformation. Fluorescence expression results showed that overly tender leaves near the leaf axis did not sustain severe damage after transformation, and water droplets formed on the surfaces due to extensive cell damage (Figure 7(C1)). Conversely, distal leaf portions were more suitable for gene gun transformation, showing no surface water droplets and higher fluorescence intensity (Figure 7(C2)). Leaf vein regions exhibited minimal fluorescent signals (Figure 7(C3)), while green leaves displayed the fewest fluorescent bright spots (Figure 7D).

The results showed that the far-pinnate leaves of the coconut were suitable recipients for genetic transformation by gene gun. However, the callus induction rate from these leaves needed to be improved to increase transformation efficiency.

## 3. Discussion

This study sought to identify suitable conditions and explant types for establishing an efficient gene gun-mediated transformation system in coconut.

### 3.1. Evaluation of Reporter Genes for Transient Detection in Coconut Transformation

We examined several factors that affect transformation success in coconut, primarily by comparing the expression of a reporter gene. These reporter genes include the most commonly used visual markers, i.e., *GUS*, eGFP, and RUBY. Among the visual markers tested, eGFP produced the strongest and most easily detectable transient fluorescence signals, suggesting that this reporter gene may be the most effective for screening transformed coconut plant material. The designed P7 vector used in our experiments contained both the eGFP and *GUS* genes, allowing for the simultaneous tracking of their expression. Our findings confirm that the eGFP reporter gene is more suitable for detecting transient transformation in coconut, as it produces clear, easily detectable fluorescent signals that can be used to identify transformed tissues in real time [15].

Endogenous β-glucuronidase (GUS or GUS-like) activity has been reported in a range of plant tissues and can produce non-specific blue staining with X-Gluc (5-bromo-4-chloro-3-indolyl β-D-glucuronide); this background activity has been documented in rice callus and seedlings and is widely discussed in other crops [21,22], so controls and staining-condition optimizations are necessary to distinguish true transgene expression from endogenous activity. In this study, a further optimization experiment was conducted for the GUS assay, which is commonly used to assess stable transformation. The effects of methanol-to-GUS ratios were investigated, and the optimal level was selected to maximize *GUS* expression. Coconut tissues exhibit endogenous GUS-like activity, which can cause nonspecific background staining. In the present study, a 4:10 ratio of methanol to GUS staining solution effectively reduced this background, allowing clearer visualization of reporter expression. This is consistent with findings in oil palm, where pretreatment of callus in hyperosmotic medium before staining improved detection sensitivity [23]. However, because GUS staining is destructive and causes tissue necrosis, it cannot be used to evaluate regeneration potential. Its application in this study was therefore limited to endpoint confirmation of transformation. Although eGFP provided a more sensitive visual readout, the fluorescence spots corresponded closely to GUS-stained regions. In contrast, eGFP enabled real-time monitoring of reporter gene expression in living tissues without impairing growth. Although eGFP provided a more sensitive visual readout, fluorescence-positive sites corresponded closely with GUS-stained regions observed in parallel samples, indicating that both markers reliably identify the same transformed cell populations. Collectively, these results demonstrate that eGFP is the preferred reporter gene for assessing transient transformation in coconut, while GUS staining remains useful for verifying transformation events only when regeneration is not required.

### 3.2. Explant Transformation Potential

The response of plant materials to transformation varies widely, depending on the transformation method and its potential for subsequent regeneration. Experimental procedures are therefore optimized by carefully selecting the appropriate plant materials for transformation and regeneration [10]. Transformation efficiency improves significantly when explants are in an active physiological state [10]. Additionally, the regenerative potential of the plant materials after transformation should be taken into consideration. The most commonly used plant materials for transformation include zygotic embryos, rachilla, leaves, and callus tissue [24]. Three main strategies have been developed to establish a complete genetic transformation system. The first strategy involves the direct transformation of callus tissue into differentiated transgenic plants through somatic embryogenesis or in-plant transformation methods, such as a gene gun or Agrobacterium-mediated transformation. The second strategy targets zygotic embryos for transformation; in this case, dedifferentiated chimeric tissues derived from transformed zygotic embryos are used to regenerate callus, which is then redifferentiated to produce homozygous transgenic plants. The third strategy involves the direct dedifferentiation of inflorescences or leaves to form callus tissue, followed by redifferentiation and plant regeneration.

Callus tissue primarily consists of thin-walled parenchyma cells capable of active cell division, making it a suitable substrate for genetic transformation [25,26]. However, calli can be classified into different types based on their histological characteristics and physical appearance. In this study, distinct transformation responses were observed among the three callus types. Spongy callus exhibited strong transient eGFP fluorescence, indicating high susceptibility to biolistic DNA delivery. However, its loose and watery texture closely resembles non-embryogenic callus as described in previous studies, which lack the capacity to regenerate somatic embryos [27,28]. In contrast, crumbly and smooth callus displayed weaker eGFP signals, possibly due to their denser tissue structure limiting microprojectile penetration. Importantly, both callus types exhibited compact morphology and nodular structures consistent with embryogenic callus characteristics reported previously [27,28]. Therefore, while spongy callus may be highly receptive to DNA delivery, crumbly and smooth callus appear to offer a more favorable balance between transformation responsiveness and morphological features associated with regeneration potential under suitable culture conditions.

To improve transformation success, physiologically active dividing calli with a thinner cell wall, which may produce a high frequency of embryogenic calli, can be optimized to enhance transformation in coconut [14]. Coconut is a recalcitrant species, and only a few studies have reported transformation efficiency ranging from 0 to 68% using Agrobacterium-mediated transformation with the red fluorescent reporter gene dsRED in calli. However, these studies did not describe the regeneration potential or the recovery of transgenic seedlings from transformed calli [15]. Therefore, further optimization of the somatic embryogenesis process is required to induce a large number of regenerable calli by adjusting plant growth regulator concentrations and culture conditions.

Histological studies revealed that cell wall modification and nuclear reorganization are critical features distinguishing embryogenic from non-embryogenic calli. Treatment with 2,4-D (2,4-Dichlorophenoxyacetic acid) induces pronounced alterations in the cell wall and activates nuclear regulatory factors in coconut tissues, corresponding to the transition toward embryogenic competency [29]. This emphasizes the importance of explant genotype and optimization of culture conditions in promoting embryogenic calli capable of complete plant regeneration.

In this study, germinated zygotic embryos exhibited numerous GFP-expressing cells in the plumule region, whereas non-germinated embryos showed no detectable signal. However, in mature plumules, GFP expression was confined to the basal region, and transformed tissues eventually failed to proliferate, indicating that transformation was not uniform and regeneration remained limited. Although excised plumule tips showed strong transient GFP fluorescence, the transformed tissue gradually formed sponge-like structures and ceased development. Bombardment of the exposed meristematic region did not yield any GFP signal, suggesting that this highly specialized tissue may be less responsive to biolistic DNA delivery under current conditions.

Despite these limitations, our results indicate that specific regions of the zygotic embryo retain strong regenerative potential following DNA delivery. Previous studies have demonstrated that zygotic embryos are effective explants for callus induction, and somatic embryoids can be regenerated after prolonged culture on embryo-induction media [30]. In addition, zygotic embryos have been widely used for germplasm conservation and exchange, with well-established in vitro propagation protocols [31]. These findings support the promise of zygotic embryos as suitable recipient tissues for continued improvement of gene gun-mediated genetic transformation in coconut.

Inflorescence and leaf blades were used as alternative explants for transformation and subsequent regeneration, and a study was conducted to assess their suitability. Rachilla of the inflorescence has previously been successfully regenerated [14], but its transformation has not been explored. Our experiment involved bombarding the P13 vector at various positions on racilla. Transient eGFP fluorescence was detected only on the surface of the immature rachilla, indicating that plasmid delivery did not reach the underlying regeneration-competent floral primordia. Therefore, rachilla should currently be considered a preliminary candidate explant, and future work must focus on improving access to floret primordia—such as by optimizing penetration strength, modifying scale barriers enzymatically, or using controlled micro-dissection techniques. Based on our observations, callus formation in our tissue culture system primarily arises from the floret sites, which contain stem-cell–enriched tissues. Thus, the transformation could be successful only if eGFP signals are observed at floret sites. Unfortunately, florets are protected by the scales along the entire length of the rachilla, which hinders the bombardment of the plasmid into the florets.

The rachilla contains developing floral primordia that are protected by tightly interlocking scales. Although longitudinal sectioning can expose inner tissues to bombardment, our results showed that this approach did not yield detectable fluorescence signals. A likely explanation is that sectioning severely damages outer cell layers, and wound-induced cell death may create a barrier that prevents plasmid penetration into viable inner tissues. To mitigate this damage, we precultured the rachilla for 10 days to allow partial tissue recovery before bombardment. Preculture has previously been shown to restore cell organization and stimulate active growth, thereby improving explant competence [24]. The studies on coconut have also demonstrated that regenerative meristematic cells gradually re-emerge during extended culture, with leaf primordia forming actively dividing cells only after ~45 days [26]. Consistent with these reports, precultivation in our study modestly improved transformation responsiveness on rachilla surfaces. However, fluorescence signals in the longitudinally sectioned regions remained scarce, indicating that recovery was incomplete and that protective scales and wound-healing responses still restrict DNA delivery to the floral primordia.

These findings suggest that while preculture enhances physiological readiness, overcoming the physical barriers associated with rachilla structure will likely require further optimization, such as adjusting the timing of tissue exposure, employing less disruptive tissue-access strategies, or modifying bombardment parameters to improve penetration depth. Addressing these limitations will be critical for establishing rachilla as a reliable explant for gene gun transformation in coconut.

### 3.3. Constraints on Transformation

The present study identified multiple biological and technical constraints that currently limit the efficiency of gene-gun transformation in coconut. Explant type played a defining role in transient reporter gene expression, and importantly, tissues showing strong transformation responsiveness did not necessarily possess traits favorable for regeneration. This underscores the need to select explant tissues that are not only accessible to microprojectile penetration but also physiologically capable of supporting subsequent cell division and embryogenic development.

Structural barriers and wound responses further restricted DNA delivery. In rachilla, the outer scale layers and wound-induced cell deposition impeded microprojectile penetration, even after preculture. Likewise, denser callus types reduced penetration depth, leading to weaker fluorescence detection. These limitations highlight the importance of improving bombardment settings (e.g., penetration force) and refining explant pretreatments to enhance tissue receptivity. High transformation efficiency ultimately depends on explants that can both acquire DNA efficiently and remain viable for recovery and potential regeneration afterward [32].

In particular, superficial transformation was evident in zygotic embryos, where transient fluorescence accumulated at the bombardment wounds instead of in the meristematic region. This indicates that although the outer tissues are permissive to DNA uptake, the inner regenerative tissues remain largely untransformed. Enhancing access to meristematic cells—either by modifying tissue exposure prior to bombardment or by improving penetration depth—will be necessary for successful regeneration of transgenic plants.

Our comparison of leaf materials further supports this conclusion. Young leaflets—especially their distal portions—displayed strong eGFP fluorescence with minimal tissue damage, indicating high responsiveness to biolistic delivery and better post-bombardment recovery. By contrast, mature green leaves showed weak or undetectable fluorescence, reflecting reduced DNA uptake capability. These results align with observations in other plant species, demonstrating that younger, actively growing tissues typically provide superior transformation outcomes [33].

## 4. Materials and Methods

### 4.1. Plant Materials

Coconut materials were obtained from the National Germplasm Nursery for Tropical Palms (Coconut Repository) in Wenchang, Hainan, China. Zygotic embryos (ZE) were collected from mature coconut fruits (12-month-old) from the 15-year-old coconut tree of the local variety ‘Hainan Tall’. The calli were induced from the plumule of ZE according to a previous method [17]. Transformation efficiency was evaluated using four types of plant materials: callus tissue, zygotic embryos, immature floral clusters, and leaves. To induce callus formation following the method described in reference [34], explants were cultured on Y3 medium solidified with 4 g/L phytagel (Solarbio, P8170, Beijing, China), supplemented with 30 g/L sucrose (Solarbio, S8270), 2.5 g/L activated charcoal (Solarbio, C7261), and 120 mg/L 2,4-dichlorophenoxyacetic acid (2,4-D) (Solarbio, D8100). Callus tissues were obtained after 6–9 months of culture. Callus tissues were classified into three morphological subtypes based on surface characteristics: spongy callus (porous and partially soft texture), rough callus (brittle surface), and smooth callus (compact, spherical appearance). All three callus subtypes were used in transient transformation assays.

Well-developed zygotic embryos were also selected as transformation recipients and further grouped according to their developmental stage and the specific excision treatment applied: (i) non-germinated embryos, (ii) germinated embryos with intact plumules, (iii) excised tips of the plumule, (iv) basal portions of the plumule capable of callus induction, and (v) the meristematic region of the plumule, exposed after removal of the outer embryonic layer. These explants were subjected to gene gun-mediated transient transformation to assess their regeneration potential following DNA delivery.

Zygotic embryos are physiologically active tissues with strong regenerative capacity and self-repair ability, making them suitable candidates for direct regeneration or callus induction after transformation. Immature floral clusters were selected as an additional explant source because they are easier to isolate than zygotic embryos and serve as high-quality materials for callus induction. For rachilla-based transformation experiments, immature rachilla segments (2–3 cm in length) were excised and precultured on Y3 medium supplemented with 30 g/L sucrose, 120 mg/L 2,4-dichlorophenoxyacetic acid (2,4-D), and 2.5 g/L activated charcoal, solidified with 4 g/L phytagel. Preculture was carried out for 10 days in darkness at 28 ± 1 °C prior to gene gun bombardment. Leaf tissues were also included due to their availability and relatively uniform cell surface, which can facilitate DNA penetration and subsequent callus formation. Collectively, callus tissue, zygotic embryos, immature rachilla, and leaves were selected for a comprehensive evaluation of transformation responsiveness.

### 4.2. Strains and Plasmids

**Figure 8:**
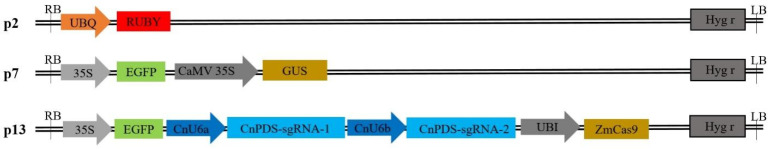
Schematic diagrams of the plasmid vectors used for gene gun-mediated transformation in coconut. P2 carries the RUBY reporter gene cassette (*CYP76AD1*, *DODA*, and GT driven by the UBQ promoter). P7 carries eGFP and GUS reporter genes driven by individual CaMV 35S promoters, respectively. P13 is a dual-target vector containing eGFP and the *CnPDS* gene driven by the CnU6 promoter.

The *E. coli* strain DH5α was transformed using plasmid vectors P2, P7, and P13 (Figure 8). The P2 vector contains the cytochrome P450 76AD1 (*CYP76AD1*), 4,5-DOPA dioxygenase (*DODA*), and glucosyltransferase (GT) driven by the UBQ (Ubiquitin) promoter, which synthesizes the red pigment called “RUBY”. The P7 vector contains the green fluorescent protein (GFP) reporter gene under the control of the 35S promoter, along with the β-glucuronidase (*GUS*) reporter gene driven by the CaMV-35S promoter. The P13 vector serves as a dual-target expression vector, containing both the GFP reporter gene (35S promoter) and the plant Phytoene desaturase (PDS) gene (*CnPDS*), which is activated by the CnU6 promoter.

### 4.3. Gene Gun-Mediated Transient Genetic Transformation

Transient genetic transformation was performed using a biolistic gene gun (GJ-1000, Ningbo Xinzhi, Ningbo, China) pressurized with 99.999% pure nitrogen gas. Gold particles were coated with the respective plasmids and used as microcarriers for DNA delivery. A single-factor experimental design was employed to determine the optimal parameters for transient expression, using coconut callus and plasmid P7 as the reporter construct.

For each transformation parameter, multiple levels were tested: Hyperosmotic pre-treatment: 0.3 M, 0.4 M, and 0.5 M mannitol; Gold particle amount per bombardment: 100 μg, 300 μg, 500 μg and 700 μg per bombardment; Plasmid DNA loading: 0.5 μg, 1.0 μg, 1.5 μg, and 2.0 μg per bombardment; Bombardment distance: 4 cm, 5 cm, 6.5 cm, 8.0 cm and 9.5 cm from the carrier membrane to the explant surface; and Nitrogen pressure: 3.5 MPa, 4.5 MPa, 7 MPa, and 8 MPa. For each treatment, approximately 35–40 callus pieces (3–5 mm in diameter) were evenly arranged on a 9 cm Petri dish. Three plates (replicates) were analyzed per parameter, and when one parameter was tested, all other parameters were maintained at the baseline conditions to ensure independent evaluation.

Each carrier membrane received gold particles at the specified amount, and transient transformation efficiency was quantified as the average number of eGFP fluorescent spots per plate, recorded 24 h after bombardment under blue excitation light. These measurements allowed direct comparison of the effects of individual parameters and supported the selection of the optimal parameter combination for subsequent analyses.

### 4.4. Pre-Treatment of the Hyperosmotic Medium and Post-Bombardment Recovery

The recipient plant materials, including callus tissues, leaves, zygotic embryos, and flower clusters, were arranged in a circular pattern (3 cm diameter) on a 9 cm Petri dish and inoculated on a solid hyperosmotic medium (MS salts supplemented with mannitol at concentrations of 0.3, 0.4, and 0.5 M) for 6 h before genetic transformation. The pre-treatment aimed to increase cell membrane permeability, making the plant material more receptive to genetic transformation. The transformed material was bombarded with a gene gun and then cultured on high-osmotic medium for an additional 16 h in the dark at 28 °C. For post-bombardment recovery, the bombarded materials were transferred to solid medium consisting of MS basal salts, 4 g/L phytagel (Solarbio, P8170), 30 g/L sucrose (Solarbio, S8270), and 2.5 g/L activated charcoal (Solarbio, C7261), and cultured for 24 h.

### 4.5. Determination of GFP Fluorescence

GFP fluorescence was examined 24 h after plasmid delivery using a Nikon SMZ18 stereomicroscope under blue excitation light (480 nm). Fluorescent images were captured using a Camera (Nikon Tokyo, Japan) mounted on the microscope to ensure clear visualization of transient GFP expression.

To enable consistent and comparable quantification across samples, fluorescence spots were analyzed using ImageJ (v1.54g) and CellProfiler (v4.2.8) with unified detection criteria. Background autofluorescence levels were established from negative control tissues, and a fixed intensity threshold (mean background intensity + 3× standard deviation) was applied to all images. Only signals exceeding this threshold and showing a minimum particle area of ≥10 µm^2^ were counted as valid GFP spots to exclude noise and scattered pixels. For each sample, six non-overlapping regions of interest (ROIs) of equal area were analyzed, and the mean number of detected spots per ROI was calculated as the transformation efficiency index. These procedures minimized variability due to tissue structure and ensured that fluorescence measurements were directly comparable across callus subtypes and other explants.

### 4.6. GUS Assay

The GUS assay was conducted using a commercially available GUS staining kit (Kualai Bo Technology Co., Ltd., Beijing, China). The calli were incubated in the GUS staining solution at 37 °C for 12 h following transformation. The samples were then bleached 2–3 times with 70% ethanol, which turned the negative control white. Blue spots indicating GUS expression were observed against the white background by visual inspection and further confirmed under a stereomicroscope. The images were captured using a Nikon camera (Nikon Corporation, Kanagawa, Japan), and fluorescence intensity was analyzed using ImageJ (v1.54g) and CellProfiler software (v4.2.8).

### 4.7. Statistical Analysis

Data analysis and visualization were performed using GraphPad Prism version 8.0.2. All experiments followed a completely randomized design with three replicates and two factors (plant material and plasmid). Results are expressed as mean ± standard deviation (SD). Mean differences were evaluated using the least significant difference (LSD) test at a significance level of *p* < 0.05.

## 5. Conclusions

This study systematically evaluated key factors affecting transient gene delivery in coconut using a biolistic transformation approach. By comparing commonly used reporter genes, we demonstrated that eGFP enables the most reliable and non-destructive visualization of transient transformation events in coconut tissues. We further showed that explant type strongly influences transformation responsiveness: young leaflets and the plumule region of germinating zygotic embryos exhibited stronger fluorescence signals than mature or structurally dense tissues, such as rachilla scales and compact callus. These findings indicate that selecting explants with accessible cell layers and active physiological status is critical to improving transient transformation performance. In addition, physical barriers such as rachilla scales and wound-induced cell layers restricted microparticle penetration, highlighting the need for continued refinement of tissue preparation and bombardment parameters. This study provides the necessary foundational parameters and explant selection information that is essential before initiating large-scale optimization of coconut regeneration post-transformation.

## Data Availability

Data are contained within the article.

## Abbreviations

The following abbreviations are used in this manuscript

PDS	Phytoene desaturase
CRISPR	Clustered regularly interspaced short palindromic repeats
GFP	Green fluorescent protein
GUS	β-glucuronidase
UBQ	Ubiquitin
SD	Standard deviation
LSD	Least significant difference
X-Gluc	5-bromo-4-chloro-3-indolyl β-D-glucuronide
2,4-D	2,4-Dichlorophenoxyacetic acid)
